# O12.5. GENETIC AND ENVIRONMENTAL PREDICTORS OF MAIN OUTCOMES IN THE DANISH HIGH RISK AND RESILIENCE STUDY - VIA 7. A STUDY OF 522 7-YEAR-OLD CHILDREN OF PARENTS WITH SCHIZOPHRENIA, BIPOLAR DISORDER OR NEITHER OF THESE DISORDERS

**DOI:** 10.1093/schbul/sby015.273

**Published:** 2018-04-01

**Authors:** Merete Nordentoft, Nicoline Hemager, Camilla Jerlang Christiani, Ditte V Ellersgaard, Aja Greve, Ditte Lou Gantriis, Birgitte Klee Burton, Katrine Spang, Kerstin von Plessen, Jens Richardt Møllegaard Jepsen, Ole Mors, Anne Amalie Thorup

**Affiliations:** 1 Mental Health Centre Copenhagen; 2 Research Unit at Mental Health Center, Capital Region of Denmark and The Lundbeck Foundation Initiative for Integrative Psychiatric Research (iPSYCH); 3 Psychosis Research Unit, Aarhus University Hospital; 4 The Lundbeck Foundation Initiative for Integrative Psychiatric Research, Psychosis Research Unit, Aarhus University Hospital; 5 Child and Adolescent Mental Health Centre, Mental Health Services Capital Region, Copenhagen; 6 Mental Health Services – Capital Region of Denmark, Child and Adolescent Mental Health Centre; 7 University Hospital Lausanne; 8 Mental Health Services – Capital Region of Denmark, Child and Adolescent Mental Health Centre, The Lundbeck Foundation Initiative for Integrative Psychiatric Research, Mental Health Services - Capital Region of Denmark, Center for Neuropsychiatric Schizophrenia Research and Center for Clinical Intervention and Neuropsychiatric Schizophrenia Research; 9 Aarhus University Hospital; 10 Child and Adolescent Mental Health Center Capital Region of Denmark

## Abstract

**Background:**

Studies of children born to parents with schizophrenia and affective disorders can allow us to study the processes preceding the manifestation of the disease, and thereby provide a possibility for identifying early amendable risk factors such as poor parenting, deviances in cognitive functioning, and early, subtle signs of psychopathology at a point where preventive intervention can be applied.

**Methods:**

The Danish High Risk and Resilience Study - VIA7 is a representative nationwide cohort study of 522 7-year-old children of parents with schizophrenia, bipolar disorder or neither of these disorders recruited during 2013–2015. The sample consists of: 202 children with a parent diagnosed with schizophrenia spectrum psychosis, 120 children with a parent diagnosed with bipolar disorder, and 200 children with neither of the parents treated in mental health services for the above diagnoses.

We have collected blood and saliva samples from the children and their parents and polygenic risk scores were calculated. We have thoroughly assessed the home environment with the instrument HOME. We have assessed main outcomes such as psychopathology, PLIKS, neurocognition and social cognition.

We will analyse the influence of genetic and environmental exposures and their interaction.

**Results:**

Generally, the children with a familial risk of schizophrenia had lower neurocognitive, social cognitive and neuromotor functioning, more child psychiatric diagnoses, and more severe symptoms compared to control children. In most comparisons, children of parents with bipolar disorder were not different from controls, but in some tests they performed poorer or had more symptoms compared to than control children.

We will present data on genetic and environmental risk factors for these outcomes

**Discussion:**

This is the largest high-risk study ever conducted. It is unique that we have access to detailed phenotyping and extensive information on environmental and genetic risk factors. Studies like this can inform about patogenesis and possibilities for future preventive interventions

